# Role of Myokines in Regulating Skeletal Muscle Mass and Function

**DOI:** 10.3389/fphys.2019.00042

**Published:** 2019-01-30

**Authors:** Jong Han Lee, Hee-Sook Jun

**Affiliations:** ^1^College of Pharmacy, Gachon University, Incheon, South Korea; ^2^Lee Gil Ya Cancer and Diabetes Institute, Gachon University, Incheon, South Korea; ^3^Gachon University Gil Medical Center, Gachon Medical and Convergence Institute, Incheon, South Korea

**Keywords:** muscular contraction, myocytes, myokines, skeletal muscle mass, muscle strength

## Abstract

Loss of skeletal muscle mass and strength has recently become a hot research topic with the extension of life span and an increasingly sedentary lifestyle in modern society. Maintenance of skeletal muscle mass is considered an essential determinant of muscle strength and function. Myokines are cytokines synthesized and released by myocytes during muscular contractions. They are implicated in autocrine regulation of metabolism in the muscle as well as in the paracrine/endocrine regulation of other tissues and organs including adipose tissue, the liver, and the brain through their receptors. Till date, secretome analysis of human myocyte culture medium has revealed over 600 myokines. In this review article, we summarize our current knowledge of major identified and characterized myokines focusing on their biological activity and function, particularly in muscle mass and function.

## Introduction

The muscle is a tissue composed of cells or fibers that produce force and movement of the body. They are primarily responsible for maintaining and changing body position, locomotion as well as the movement of internal organs. Different types of muscles perform different functions according to their location and type. Skeletal muscles are one of the most dynamic tissues involved in voluntary contraction according to command (Frontera and Ochala, 2015; Noto and Edens, 2018). They comprise approximately 40% of the total body weight (Frontera and Ochala, 2015; Noto and Edens, 2018). In contrast, cardiac and smooth muscles are associated with involuntary contraction without awareness (Frontera and Ochala, 2015; Hafen and Burns, 2018; Noto and Edens, 2018). Smooth muscles are found throughout the body and tightly regulate many of the body subsystems implicated in maintaining survival (Hafen and Burns, 2018).

Myokines are cytokines or peptides synthesized and released by myocytes in muscle tissue in response to muscular contractions (Pedersen et al., 2007). The term “myokine” was first introduced by a Swedish scientist, Bengt Saltin, in 2003 (Pedersen et al., 2003). Myokines are implicated in the autocrine regulation of metabolism in muscles as well as in the para/endocrine regulation of other tissues and organs including the adipose tissue, liver, and brain (Carson, 2017) through their receptors. Since myostatin was first identified as a myokine in 1997, secretome-based analysis of human myocyte culture medium has revealed over 600 myokines till date (Gorgens et al., 2015). However, the majority of these myokines are still not sufficiently characterized. Only few of them have been studied for their biological activity and function and have provided some clear evidence as being released directly from muscle contraction. Moreover, studies potentially associated with muscle atrophy barely exist. Understanding the biological and physiological roles of these myokines in skeletal muscle atrophy or weakness is important and valuable to find novel targets for therapeutic intervention.

In this review, we summarize our current knowledge focusing on myokines released directly by muscle contraction and their potential roles associated with skeletal muscle mass and function.

## Myostatin

Myostatin, growth differentiation factor 8, was the first identified myokine in 1997 by Se-Jin Lee and his colleagues (McPherron et al., 1997). It is encoded by the myostatin gene and is known as a highly conserved member of the TGF beta protein family (McPherron et al., 1997). It is abundantly expressed in skeletal muscles, but is also expressed to a lesser extent in cardiac muscles and fat tissues (McPherron et al., 1997; Sharma et al., 1999). Myostatin levels in the plasma of healthy young men has been shown to significantly decrease within 24 h post-exercise when compared to pre-exercise and has also been shown to positively correlate with plasma IL-6 (Kazemi, 2016). In contrast, serum myostatin has been shown to increase in patients with spinal cord injury after aerobic exercise (Han et al., 2016). Although heavily contradicting reports exist on both sides, circulating myostatin shows an obvious increase in females rather than males during sarcopenia (Bergen et al., 2015), and a decrease in cancer-cachexia (Loumaye and Thissen, 2017) and generic neuromuscular disease patients (Awano et al., 2008; Anaya-Segura et al., 2015; Burch et al., 2017).

The effects of myostatin are mediated through the activin type IIB receptor (ActRIIB), which is expressed ubiquitously (Pistilli et al., 2011; Amthor and Hoogaars, 2012). The downstream mediators of myostatin, Smad2 and Smad3, are phosphorylated and form a complex with Smad4. This complex in turn stimulates FoxO-dependent transcription and regulates the transcription of genes associated with the proliferation and differentiation in skeletal muscle precursor cells as well as protein degradation pathways (such as the ubiquitin-proteasome processes, and autophagy) in mature myofibers (Burks and Cohn, 2011; Han et al., 2013). In addition, myostatin-mediated Smad signaling activation inhibits protein synthesis in muscle tissues by suppressing the Akt-mediated mTOR signaling pathway (Han et al., 2013). Functionally, myostatin is a negative regulator of muscle growth thereby leading to inhibition of myogenesis through muscle cell differentiation and growth (McPherron et al., 1997). Animals blocking myostatin activity with substance show significantly increased muscle mass (myofiber hypertrophy rather than hyperplasia) (Morvan et al., 2017). In myostatin-knockout mice, the muscle mass is approximately twice increased compared to that in normal mice (McPherron et al., 1997). In humans, individuals with mutations in both copies of the myostatin gene showed significantly increased muscle mass and muscle strength compared to that observed in normal individuals (Schuelke et al., 2004). Growing evidence indicates that increasing myostatin and its analog activin A contribute to the incidence of muscle atrophy (Morvan et al., 2017). Thus, myostatin is considered a promising target molecule for the treatment of muscle wasting. In the past two decades, several agents, such as follistatin (myostatin antagonist), and selective antibody-based approaches targeting ActR-IIB, myostatin, and activin A were developed to antagonize/suppress myostatin signaling. These molecules were evaluated under various pathological conditions such as muscular wasting or atrophy. For example, a myostatin antibody, MYO-029/stamulumab, was tested in broad muscle dystrophic models, including Becker’s muscular dystrophy (BMD) and facioscapulohumeral dystrophy, but failed to show clinical efficacy in elevating muscle strength (Leung et al., 2015). Overexpression of the follistatin isoform, FS344, using an AVV vector showed improved ambulation in patients with BMD and inclusion body myositis (Al-Zaidy et al., 2015; Mendell et al., 2015; Mendell et al., 2017). However, so far none of these treatments have proven to be clinically sufficient as shown in Table 1 (Cohen et al., 2015; Mariot et al., 2017). There are still obstacles (such as lack of target specificity and potential clinical toxicities) to overcome for their use in human patients. In addition, a recent study showed that activin A prominently regulates muscle mass in primates than does myostatin in rodents (Busquets et al., 2012; Cohen et al., 2015), suggesting that targeting myostatin alone may not be sufficient to treat muscle atrophy in humans.

**Table 1:** Summary of current trials of myostatin-activin pathway inhibitors.

Mechanism of action	Drug/compound	Test pathological models	Current states
Myostatin antibody	MYO-029/Stamulumab PF-06252616/Domagrozumab 2495655-Ly/Landogrozumab REGN-1033/Trevogrumab	Muscle dystrophy (BMD, FSHD, and LGMD) Duchenne muscular dystrophy Cancer cachexia Sarcopenia	Dropped Dropped Ongoing Ongoing


Myostatin peptide	AMG-745/PINTA-745	Chronic kidney disease	Dropped
Activin A antibody	REGN-2477	Healthy subjects	Ongoing
ActRllb-FC (myostatin decoy receptor)	ACE-031	Duchenne muscular dystrophy	Dropped
Anti-myostatin adnectin	BMS-986089	Ambulatory boys with DMD	Ongoing
ActRIIB antibody	BYM-338/Bimagrumab	Cancer cachexia Sarcopenia Type 2 diabetes	Ongoing
Myostatin antagonist	Follistatin isoform FS344	Becker muscular dystrophy	Ongoing


*ACE-031, Ramatercept; REGN, Trevogrumab; BMD, Becker’s muscular dystrophy; FSHD, Facioscapulohumeral dystrophy; LGMD, limb-girdle muscular dystrophy*.

## Irisin

Irisin is a cleaved form of Fibronectin type III domain-containing protein 5 (FNDC5), which was simultaneously discovered by two independent groups in 2002 (Teufel et al., 2002; Colaianni et al., 2014) It was first reported as a potential mediator of the beneficial effect of exercise (Raschke et al., 2013b). Initially, the expression of PGC1α in muscle stimulates FNDC5 expression, which drives brown fat-like development of white fat cells named beige cells and increases thermogenesis (Bostrom et al., 2012). Although exercise-induced increase in the level of irisin in the blood is heavily debated (Pekkala et al., 2013), many reports have continuously shown an increase in FNDC5 mRNA expression upon exercise in rodent models (Dun et al., 2013; Roberts et al., 2013) and humans (Huh et al., 2012; Lecker et al., 2012), thus triggering renewed interest in exercise-induced myokines. In line with these observations, expression of mitochondrial-specific transcription factors, such as PGC-1α and mitochondrial transcription factor A, increases in C2C12 myotubes exposure to recombinant irisin for 24 h. They are all involved in elevated mitochondrial content and oxygen consumption (Vaughan et al., 2015). Moreover, irisin and myostatin are inversely secreted from skeletal muscles after physical exercise (MacKenzie et al., 2013), thereby suggesting its potential myogenic role. Reza et al. reported that irisin induced skeletal muscle hypertrophy and attenuated denervation-induced atrophy by activating IL-6 signaling in rodents (Reza et al., 2017). The effects of irisin on hypertrophy were shown to be established by the activation of muscle satellite cells and elevation of protein synthesis (Reza et al., 2017). This study substantially opened up potential research avenues on irisin with respect to muscle atrophy. Moreover, a latest study showed that circulating irisin levels were lower in women with postmenopausal sarcopenia when compared to those with pre-sarcopenia and that they negatively correlated with the quadricep cross-sectional area (Park et al., 2018), suggesting that irisin may also function as a potential pro-myogenic factor in human pathological conditions. Further studies are needed to reveal the biological effects of human irisin and the underlying mechanism in human skeletal muscles.

## IL-6

Interleukin 6 (IL-6) was identified in 2000 and is the most studied myokine (Steensberg et al., 2000; Pedersen and Febbraio, 2008). It is secreted from muscles into the blood vessel in response to muscle contractions (Pedersen and Febbraio, 2008), by which skeletal muscles communicate with central and peripheral organs (Pedersen et al., 2003). The circulatory level of IL-6 is affected by both the duration and intensity of muscle contraction in humans (Steensberg et al., 2000; Helge et al., 2003). Interestingly, IL-6 is highly produced and released after post-exercise while insulin action is enhanced. However, IL-6 is also associated with obesity and insulin resistance (Pedersen and Febbraio, 2008). IL-6 has an insulin-like effect on glucose metabolism. IL-6 increases insulin-stimulated glucose disposal in humans as well as glucose uptake and fatty acid oxidation *in vitro* through AMP-activated protein kinase and PI3K-Akt signaling pathways (Al-Khalili et al., 2006; Carey et al., 2006). Individuals with spinal cord injury (SCI) are prone to develop metabolic diseases due to the lack of exercise-related IL-6 response, suggesting that IL-6 plays a pivotal role in regulating glucose homeostasis (Kouda et al., 2012).

On the other hand, the role of IL-6 on muscle atrophy seems to be a negative effect rather than a beneficial effect. Increased circulating angiotensin II (AngII) reduces lean body mass in chronic kidney disease. In mice, AngII infusion resulted in increased circulating IL-6 and its hepatic production, suggesting that AngII-induced inflammation might be a trigger for muscle loss (Zhang et al., 2009). In contrast, AngII-induced muscle atrophy was suppressed in IL-6-deficient mice (Zhang et al., 2009). IL-6 is overproduced in patients with Duchenne muscular dystrophy and in muscles of the mdx animal model. Inhibition of IL-6 activity with an interleukin-6 receptor (Il-6r) neutralizing antibody attenuates the dystrophic phenotype, severe muscle degeneration, inflammation, as well as accumulation of non-functional fat and fibrotic tissues (Wada et al., 2017). In addition, pharmacological inhibition of IL-6 activity in mdx male mice inhibits anti-inflammatory responses and improvement in muscle repair (Pelosi et al., 2015). Therefore, inhibition of IL-6 might be beneficial for preventing muscle loss.

## Brain-Derived Neurotrophic Factor

Brain-derived neurotrophic factor (BDNF) is the second member of the neurotrophin family of growth factors, which regulates neuronal survival, plasticity, growth, and death through tropomyosin-related kinase receptor B (TrkB). It was for the first time purified from pig brain in 1982 (Barde et al., 1982). After 11 years, the BDNF gene was identified by two independent groups (Metsis et al., 1993; Binder and Scharfman, 2004). Initially, BDNF has been studied mostly in relation with nervous system development and function (Clow and Jasmin, 2010). However, the expression of several neurotrophin receptors is identified in skeletal muscles, thus implicating the certain role of BDNF. Indeed, Chevrel et al. (2006) reported that BDNF is differentially expressed in skeletal muscles according to physiological or pathological conditions. In adult skeletal muscles, BDNF is also expressed in muscle satellite cells (Mousavi et al., 2004) and is upregulated in muscle injury followed by the activation and proliferation of satellite cells, suggesting that BDNF might play an important role in mediating the satellite cell response to muscle injury (Omura et al., 2005). Jasmin et al. showed that BDNF substantially regulates satellite cell differentiation and skeletal muscle regeneration, by using BDNF null and muscle-specific BDNF KO mice (Clow and Jasmin, 2010). These results indicate that BDNF might be involved in the regulation of damaged muscles. Although there are many studies associated with the role of BDNF in muscle development and function, there is no clear evidence indicating that it is a myokine. In fact, the effect of muscle contraction on circulating BDNF levels is controversial. Some studies have reported no change in serum BDNF right after either acute or chronic exercise. On the other hand, several studies have shown that circulating BDNF increases with physical exercise (Ferris et al., 2007; Yarrow et al., 2010; Pereira et al., 2018). In skeletal muscle cells, BDNF mRNA expression is increased by contraction and increased fat oxidation through activation of AMP-activated protein kinase (Matthews et al., 2009). Overall, these studies suggest that muscle-derived BDNF is important for regulating muscle regeneration right after muscle injury. However, many key questions on the biological functions of BDNF in skeletal muscles remain unresolved. A major issue would be to elucidate the mechanism by which BDNF regulates satellite cell differentiation and skeletal muscle regeneration, and in which BDNF substantially recover muscle strength and function. Manipulating BDNF may thus represent an important therapeutic tool for alleviating dystrophic muscle atrophy.

## IL-15

Interleukin-15 (IL-15) is a cytokine with a structure similar to interleukin-2 (IL-2). It was discovered by two different research groups in 1994 and was characterized as a T cell growth factor (Steel et al., 2012). Later on, several studies showed that IL-15 is accumulated in the muscles as a result of regular exercise training, indicating that it is a myokine (Pedersen, 2011; Tamura et al., 2011; Brunelli et al., 2015). Moreover, IL-15 mRNA expression is upregulated along with myoblast differentiation (Pedersen and Febbraio, 2008). Supportively, several studies showed that exogenously treated IL-15 or IL-15 overexpression promotes myoblast differentiation and increases muscle mass in the mouse C2 skeletal myogenic cell line (Quinn et al., 1995, 2002). In rats with cancer cachexia, IL-15 treatment attenuates skeletal muscle wasting by suppressing protein degradation through inhibition of the ATP-dependent ubiquitin proteolytic pathway (Carbo et al., 2000). IL-15 administration was found to improve diaphragm strength with increased muscle fiber cross-sectional area and decreased collagen accumulation in dystrophic mdx mice (Harcourt et al., 2005). In contrast, systemic infusion of IL-15 induces muscle atrophy in skeletal muscles of rodents (Pistilli and Alway, 2008). IL-15 treatment increased the glucose uptake in skeletal muscle cells via activation of the Jak3/STAT3 signaling pathway (Krolopp et al., 2016) or the AMPK signaling pathway (Gray and Kamolrat, 2011). In addition, Quinn L et al. and coworkers reported that IL-15 transgenic mice exhibited increased fat oxidation, energy expenditure and running endurance even with lower muscle mass compared to that in wild type mice. Interestingly, these mice also expressed troponin I and myosin heavy chain mRNA isoform indicating the conversion of muscles to a more oxidative phenotype (Quinn et al., 2013; Chalkiadaki et al., 2014). Collectively, the above controversial reports indicate that IL-15 acts differently according to the normal and pathological conditions. Thus, further studies should be focused on clarifying the different factors influencing the varying roles of IL-15 between different physiological conditions.

## Myonectin (CTRP15)

Myonectin is a myokine belonging to the C1q/TNF-related protein (CTRP) family, and was discovered by Seldin et al. (2012). It is a novel nutrient-responsive myokine secreted from skeletal muscles (Seldin et al., 2012; Peterson et al., 2014). Myonectin is released into the blood stream by muscular contraction, and is functionally similar to insulin as it promotes fatty acid uptake into cells by increased expression of fatty acid transport genes (CD36, FATP1, Fabp1, and Fabp4) (Seldin and Wong, 2012; Seldin et al., 2012). In the mouse liver and cultured hepatocytes, recombinant myonectin treatment suppresses starvation-induced autophagy by inhibiting LC3-dependent autophagosome formation, p62 degradation, and other autophagy-related genes expression. Furthermore, the ability of myonectin to suppress autophagy is abolished by inhibition of the PI3K/Akt/mTOR signaling pathway (Seldin et al., 2013). Autophagy is considered to be a mechanism that induces muscle atrophy (Bonaldo and Sandri, 2013). In addition, the PI3K/Akt signaling pathway is involved in anabolic responses in the body. Therefore, these observations indicate that myonectin may play an important role in increasing muscle mass by elevating protein synthesis and inhibiting protein degradation. On a related note, mitochondrial content in muscles is an important determinant for muscle type and function. Myonectin is remarkably increased following depletion of mtDNA and increases glucose uptake and fatty acid oxidation through activation of the AMPK signaling pathway in rat skeletal myocytes (Park et al., 2009; Lim et al., 2012). Interestingly, oxidative, slow-twitch muscle fiber types express a higher level of myonectin than does glycolytic, fast-twitch muscle fiber types, suggesting that it may be involved in mitochondrial biogenesis and sensing cellular energy state (Seldin and Wong, 2012). However, there is no study associated with its biological function and mechanism on muscle mass and muscle mitochondrial biogenesis in normal physiology and in the diseased state.

## Decorin

Decorin is small leucine-rich proteoglycan identified as a myokine by Kanzleiter et al. (2014). It is secreted in skeletal muscles during muscular contraction and plays an important role in muscle growth. Decorin directly binds to and inactivates myostatin (a potent inhibitor of muscle growth) in a zinc-dependent manner, and inhibits its anti-myogenic effects (El Shafey et al., 2016). *In vivo* over-expression of Decorin in murine skeletal muscles promotes expression of the pro-myogenic factor Mighty (Marshall et al., 2008). Mighty is ubiquitously expressed but appears to be negatively regulated by myostatin in skeletal muscles. Decorin overexpression increases the expression of Myod1 and follistatin, whereas it reduces the muscle-specific ubiquitin ligases atrogin1 and MuRF1 (Marshall et al., 2008). Thus, Decorin might act as a myogenic factor and might be a possible therapeutic target for the treatment of muscle wasting.

## Fibroblast Growth Factor (FGF) 21

Fibroblast growth factors (FGFs) are signaling proteins with diverse biological functions in development and metabolism. FGFs are classified as para, intra, and endocrine according to their action manners. Paracrine FGFs mostly function as local signaling molecules in developmental processes whereas intracrine FGFs mainly serve as intracellular molecules in neuronal processes (Itoh and Ornitz, 2011). FGF21 functions as endocrinal hormone-like or local signaling molecules in metabolism. FGF21 does not have proliferative activity as other paracrine and endocrine FGFs family and is only associated with metabolism (Itoh, 2014). FGFs activate several intracellular signaling pathways including phosphatidylinositol 3-kinase (PI3K)/serine-threonine protein kinase AKT, signaling transducer and activator of transcription (STAT), mitogen activation protein kinase (MAPK), and phosphoinositide phospholipase C (PLC) γ (Itoh, 2014). Specifically, FGF21 acts through FGF receptor 1c with β-Klotho as a cofactor. Skeletal muscle-specific Akt1 transgenic mice showed skeletal muscle fiber hypertrophy with increasing Fgf21 expression in the muscle and in serum indicating that FGF21 plays an important role in regulating muscle mass (Izumiya et al., 2008). In addition, FGF21 expression is coupled to mitochondrial dysfunction and insults of various stresses in skeletal muscles. Autophagy deficiency and subsequent mitochondrial dysfunction elevates the level of FGF21 as a myokine, thereby protecting against diet-induced obesity and insulin resistance (Kim et al., 2013; Keipert et al., 2014). In cultured myoblasts, mitochondrial complex inhibitor treatment increased expression by promoting the binding of activating transcription factor 2 (ATF2) for the promoter region of the Fgf21 gene (Ribas et al., 2014). Moreover, in human brain vascular smooth muscle cells, FGF21 protects against angiotensin II-induced cerebrovascular aging by elevating mitochondrial biogenesis (Wang et al., 2016). All the above studies suggest that FGF21 may be potentially involved in switching muscle type and regulating mitophagy, thereby regulating muscle mass and function. Thus, targeting FGF21 might be an attractive approach to treat mitochondrial-based myopathy and muscle dysfunction.

## Secreted Protein Acidic and Rich in Cysteine (SPARC)

Aoi et al. (2013) reported SPARC/osteonectin as a novel myokine, which is released from the skeletal muscles of both humans and mice after exercise, even though it was identified earlier (Aoi et al., 2013). Exercise-stimulated SPARC secretion was shown to inhibit colon tumorigenesis by enhancing apoptosis in colon cancer cells (Aoi et al., 2013). SPARC was also shown to be upregulated in inherited and idiopathic muscle wasting diseases such as Duchenne muscular dystrophy and congenital muscular dystrophy (Jorgensen et al., 2009). SPARC overexpression almost completely abolished myogenic differentiation in the muscle progenitor cell line, C2C12 (Petersson et al., 2013). Thus, SPARC may play a certain functional role in the repair of muscle damage in muscle satellite cells. However, very limited studies associated with the role of myokines are currently available. Further studies need to first determine and address the expression profile and role of SPARC in muscle development and regeneration. The underlying signaling pathways also need to be studied in detail.

## Conclusion

**FIGURE 1:**
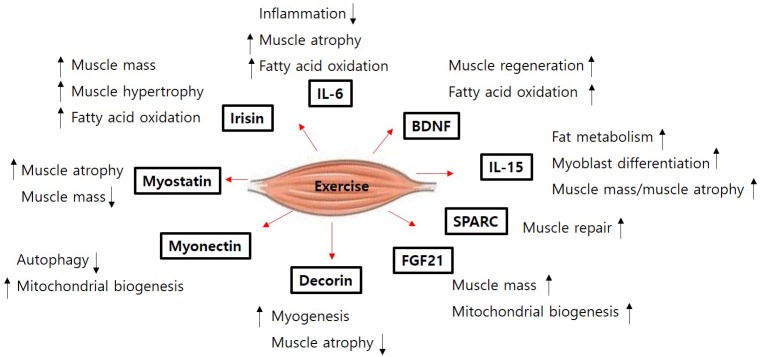
The function of muscle contraction-induced myokines. The figure shows the selected the functions for each myokines released from muscle contraction (exercise) in muscle. BDNF, brain-derived neurotrophic factor; FGF21, fibroblast growth factor 21; SPARC, secreted protein acidic and rich in cysteine; IL, interleukin.

**FIGURE 2:**
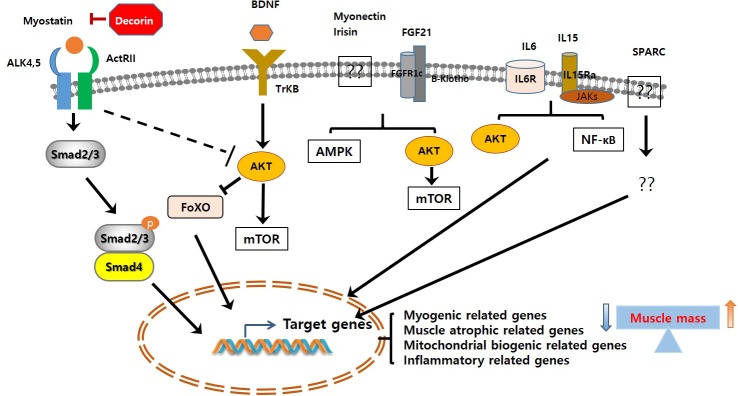
Signaling pathways of muscle contraction-induced myokines. The myokine-mediated signaling pathways lead to its target genes expression, which in turn regulate muscle cell proliferation, differentiation, and growth. It ultimately increases/decreases muscle mass. ALK, activin receptor-like kinase; ActRIIB, activin type IIB receptor; BDNF, brain-derived neurotrophic factor; TrKB, tropomyosin-related kinase receptor B; FGF21, fibroblast growth factor 21; SPARC, secreted protein acidic and rich in cysteine.

Skeletal muscle atrophy is an emerging medical problem worldwide owing to increasing elderly populations and various classical reasons including genetic mutation, disease-derived cachexia, and accidents. However, although our understanding of molecular mechanisms regulating muscle atrophy/muscle weakness has substantially progressed, there is no specific treatment for muscle atrophy. Recently, a number of myokines have been identified through secretome analysis and some have proven to be very informative while searching for novel myokines (Raschke et al., 2013a; Hartwig et al., 2014; Grube et al., 2018). However, the majority of myokines are not still sufficiently characterized with regard to their biological activity and function. Only few myokines have been restrictively characterized (Figure 1) and their potential signaling pathways, implicated in muscle cell proliferation, differentiation, and growth in order to maintain muscle mass, muscle strength, and function, been identified (Figure 2). Therefore, it is important to better understand their precise role and function on skeletal muscles under normal physiological and pathophysiological conditions. Targeting novel myokines for either increasing or suppressing their functional activity in certain pathological states could be an attractive novel therapeutic tool for combating skeletal muscle atrophy.

## Author Contributions

Both authors conceived and wrote the manuscript and approved it for publication.

## Conflict of Interest Statement

The authors declare that the research was conducted in the absence of any commercial or financial relationships that could be construed as a potential conflict of interest.
